# Citation bias favoring positive clinical trials of thrombolytics for acute ischemic stroke: a cross-sectional analysis

**DOI:** 10.1186/s13063-016-1595-7

**Published:** 2016-09-28

**Authors:** Benjamin S. Misemer, Timothy F. Platts-Mills, Christopher W. Jones

**Affiliations:** 1 Department of Emergency Medicine, Cooper Medical School of Rowan University, One Cooper Plaza, Suite 152, Camden, NJ 08103 USA; 2 Department of Emergency Medicine, University of North Carolina, Chapel Hill, 170 Manning Drive, CB#7594, Chapel Hill, NC 27599 USA

**Keywords:** Stroke, Bias, Tissue plasminogen activator, Citation bias

## Abstract

**Background:**

Citation bias occurs when positive trials involving a medical intervention receive more citations than neutral or negative trials of similar quality. Several large clinical trials have studied the use of thrombolytic agents for the treatment of acute ischemic stroke with differing results, thereby presenting an opportunity to assess these trials for evidence of citation bias. We compared citation rates among positive, neutral, and negative trials of alteplase (tPA) and other thrombolytic agents for stroke.

**Methods:**

We used a 2014 Cochrane Review of thrombolytic therapy for the treatment of acute stroke to identify non-pilot, English-language stroke trials published in MEDLINE-indexed journals comparing thrombolytic therapy with control. We classified trials as positive if there was a statistically significant primary outcome difference favoring the intervention, neutral if there was no difference in primary outcome, or negative for a significant primary outcome difference favoring the control group. Trials were also considered negative if safety concerns supported stopping the trial early. Using Scopus, we collected citation counts through 2015 and compared citation rates according to trial outcomes.

**Results:**

Eight tPA trials met inclusion criteria: two were positive, four were neutral, and two were negative. The two positive trials received 9080 total citations, the four neutral trials received 4847 citations, and the two negative trials received 1096 citations. The mean annual per-trial citation rates were 333 citations per year for positive trials, 96 citations per year for neutral trials, and 35 citations per year for negative trials. Trials involving other thrombolytic agents were not cited as often, though as with tPA, positive trials were cited more frequently than neutral or negative trials.

**Conclusions:**

Positive trials of tPA for ischemic stroke are cited approximately three times as often as neutral trials, and nearly 10 times as often as negative trials, indicating the presence of substantial citation bias.

**Electronic supplementary material:**

The online version of this article (doi:10.1186/s13063-016-1595-7) contains supplementary material, which is available to authorized users.

## Background

Stroke is the second most frequent cause of death globally [1]. Early thrombolytic treatment with alteplase (tPA) is one of the few therapies to be identified that might improve stroke outcomes, and existing guidelines overwhelmingly favor the use of tPA in selected patients [2–4]. However, the use of tPA is controversial because some clinical trials have shown benefit, some have shown no effect, and some have shown harm [5].

### Importance

One factor that might influence the translation of evidence into clinical practice is citation bias, the selective citation of papers whose results support authors’ preconceived opinions regarding treatment efficacy. Because citation bias often results in the disproportionate citation of studies with statistically significant or “positive” results, it may result in a distortion of the perceived efficacy of medical treatments within the published scientific literature [6]. Prior efforts to characterize the presence of citation bias in the medical literature have observed this form of bias among studies of therapeutic interventions, with approximately twice as many citations for studies with statistically significant results [7], but not among a broader range of study types [8].

### Goals of this investigation

We assessed for evidence of citation bias among clinical trials of tPA and other thrombolytic agents in the treatment of acute ischemic stroke.

## Methods

### Study design

This was a cross-sectional study of trials assessing intravenously administered thrombolytic therapy as an intervention for acute ischemic stroke.

### Trial selection

We identified eligible trials using a 2014 Cochrane Systematic Review of thrombolytics for ischemic stroke [9]. The investigators of this systematic review utilized a comprehensive strategy to search for trials of thrombolytic agents for the treatment of ischemic stroke by searching MEDLINE, EMBASE, the Cochrane Stroke Group Trials Register, relevant conference proceedings, manuscript reference lists, and by contacting pharmaceutical companies and investigators known to participate in thrombolytic research. Our primary analysis included trials which compared tPA to placebo or open control for the treatment of ischemic stroke. We focused primarily on tPA, as it is currently the only Food and Drug Administration-approved thrombolytic agent for acute stroke. A secondary analysis included trials comparing thrombolytic agents other than tPA (streptokinase, urokinase, prourokinase, and desmotoplase) to controls. Trials were excluded if they were not published in English, not published in a MEDLINE-indexed journal, or if they were pilot studies with fewer than 50 participants, as we hypothesized that these characteristics would substantially impact citation counts.

### Trial classification

Trials were classified as positive if the defined primary outcome met investigator-established criteria for statistical significance favoring the intervention arm, neutral if there was no significant primary outcome difference, and negative if the primary outcome showed a statistically significant effect favoring the control arm, or if safety concerns supported halting the trial early.

### Outcomes and analysis

We collected annual citation counts through December 2015 using Scopus. Citation data were updated as of 6 January 2016. We compared cumulative citation counts among positive, neutral, and negative trials graphically. Annual citation rates were calculated by dividing the total number of citations by the time elapsed from the earlier of the print or online publication dates and the end of 2015. Jadad quality scores were calculated by two study authors for each of the included trials; discrepancies were resolved by consensus [10]. We calculated the Pearson correlation coefficient to assess the relationship between trial size and annual citation rate. Data were collected and analyzed using Excel (Microsoft Corp., Redmond, WA, USA) and PASW version 18.0 (SPSS Inc, Chicago, IL, USA). The STROBE Statement checklist for observational studies is included as an additional file (see Additional file 1).

## Results

### Trial selection

Of the 27 thrombolytic trials identified in the 2014 Cochrane Review, 12 did not meet inclusion criteria: one was not published in English; three were not indexed to MEDLINE; and eight were pilot studies. The eight included tPA trials were published between 1995 and 2012, six in major general medical journals and two in specialty neurology journals. Seven trials investigated thrombolytic agents other than tPA (Table 1).

**Table 1:** Publication characteristics and citation rates for included trials of thrombolytics for stroke

Trial	Year published	Patients enrolled	Location	Journal	Intervention	Jadad score	Result	Total citations	Citations per year
NINDS [11]	1995	624	US	*NEJM*	IV tPA	5	Positive	6622	330
ECASS III [12]	2008	821	Europe	*NEJM*	IV tPA	5	Positive	2458	335
ECASS [13]	1995	620	Europe	*JAMA*	IV tPA	5	Neutral	2138	106
ECASS II [14]	1998	800	Europe, Australia, NZ	*Lancet*	IV tPA	5	Neutral	1768	102
EPITHET [15]	2008	101	Australia, NZ, Belgium, UK	*Lancet Neurology*	IV tPA	5	Neutral	561	72
IST-3 [16]	2012	3035	Europe, Canada, Mexico, Australia	*Lancet*	IV tPA	3	Neutral	380	104
ATLANTIS B [17]	1999	613	US, Canada	*JAMA*	IV tPA	5	Negative	820	51
ATLANTIS A [18]	2000	142	US	*Stroke*	IV tPA	5	Negative	276	18
PROACT II [19]	1999	180	US, Canada	*JAMA*	IA pro-urokinase	3	Positive	2287	142
DIAS [20]	2005	104	Europe, Australia, Singapore	*Stroke*	IV desmoteplase	5	Positive	749	69
DIAS-2 [21]	2009	193	Australia, Europe, US, Canada, China, Singapore	*Lancet Neurology*	IV desmoteplase	5	Neutral	332	49
MELT [22]	2007	114	Japan	*Stroke*	IA urokinase	3	Neutral	282	34
ASK [23]	1996	340	Australia	*JAMA*	IV streptokinase	5	Negative	328	17
MAST-E [24]	1996	310	Europe	*NEJM*	IV streptokinase	5	Negative	421	22
MAST-I [25]	1995	622	Europe	*Lancet*	IV streptokinase	3	Negative	551	27

*IA* intra-arterial, *IV* intravenous, *JAMA* Journal of the American Medical Association, *NEJM* New England Journal of Medicine, *NZ* New Zealand, tPA alteplase, *US* United States, *UK* United Kingdom

### Main results

The eight included tPA trials consisted of two reporting a statistically significant benefit for the primary outcome (positive trials) [11, 12], four that completed enrollment but showed no benefit based on the primary outcome (neutral trials) [13–16], and two that were stopped early due to Safety Committee concerns (negative trials) [17, 18].

**Fig. 1:**
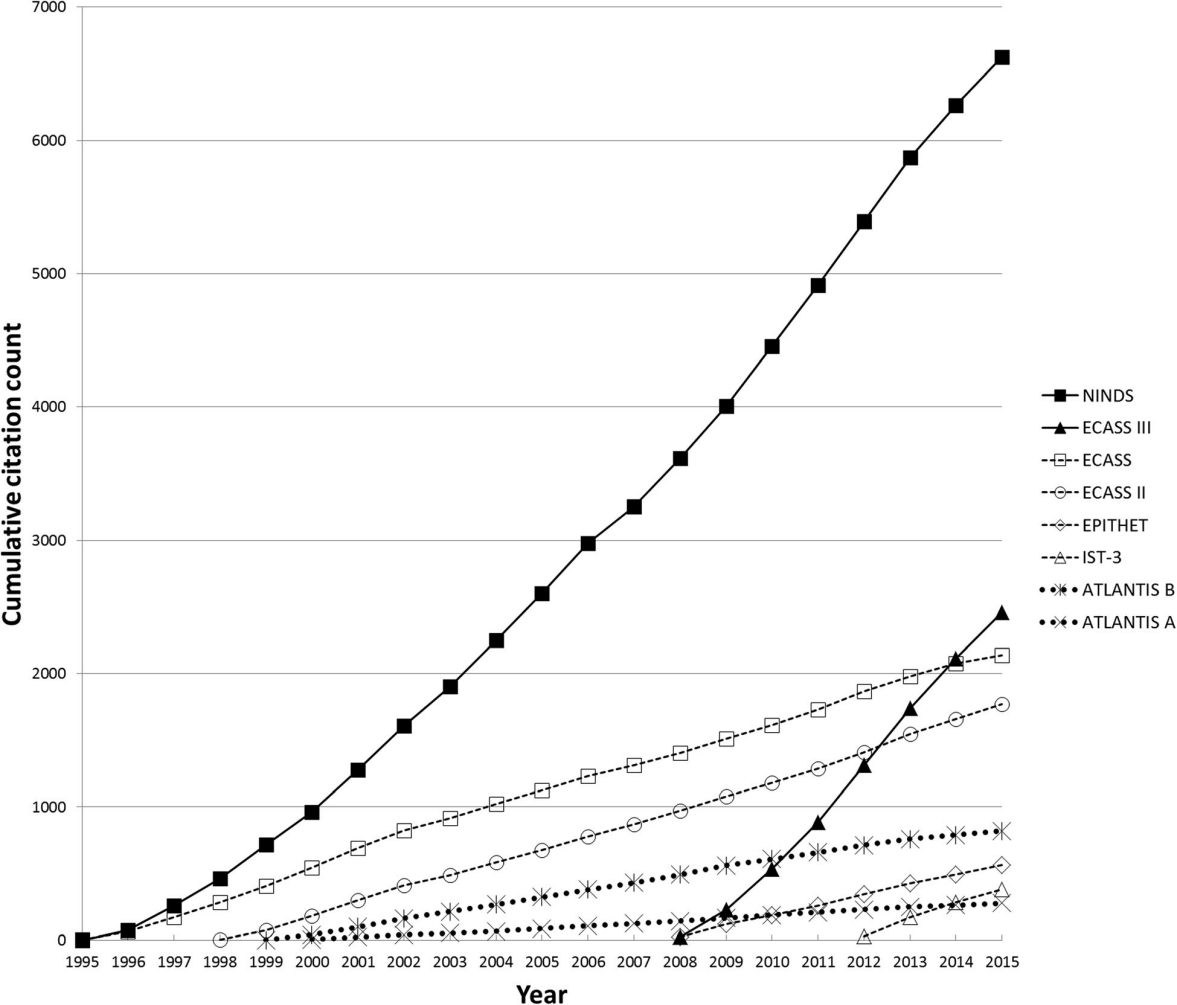
Citation rates of alteplase (tPA) trials for acute stroke. Cumulative citation counts over time for positive (*solid lines*), neutral (*dashed lines*), and negative (dotted lines) randomized controlled trials of tPA for acute ischemic stroke

The two positive trials received 9080 total citations; the four neutral trials received 4847 citations; and the two negative trials received 1096 citations. Mean annual per-trial citation rates were 333 per year for the positive trials, 96 per year for the neutral trials, and 35 per year for the negative trials. Figure 1 shows cumulative citation counts for individual trials. Visual analysis of these results shows that heterogeneity with respect to the enrollment windows for the included trials does not explain the observed differences in citation rates.

**Fig. 2:**
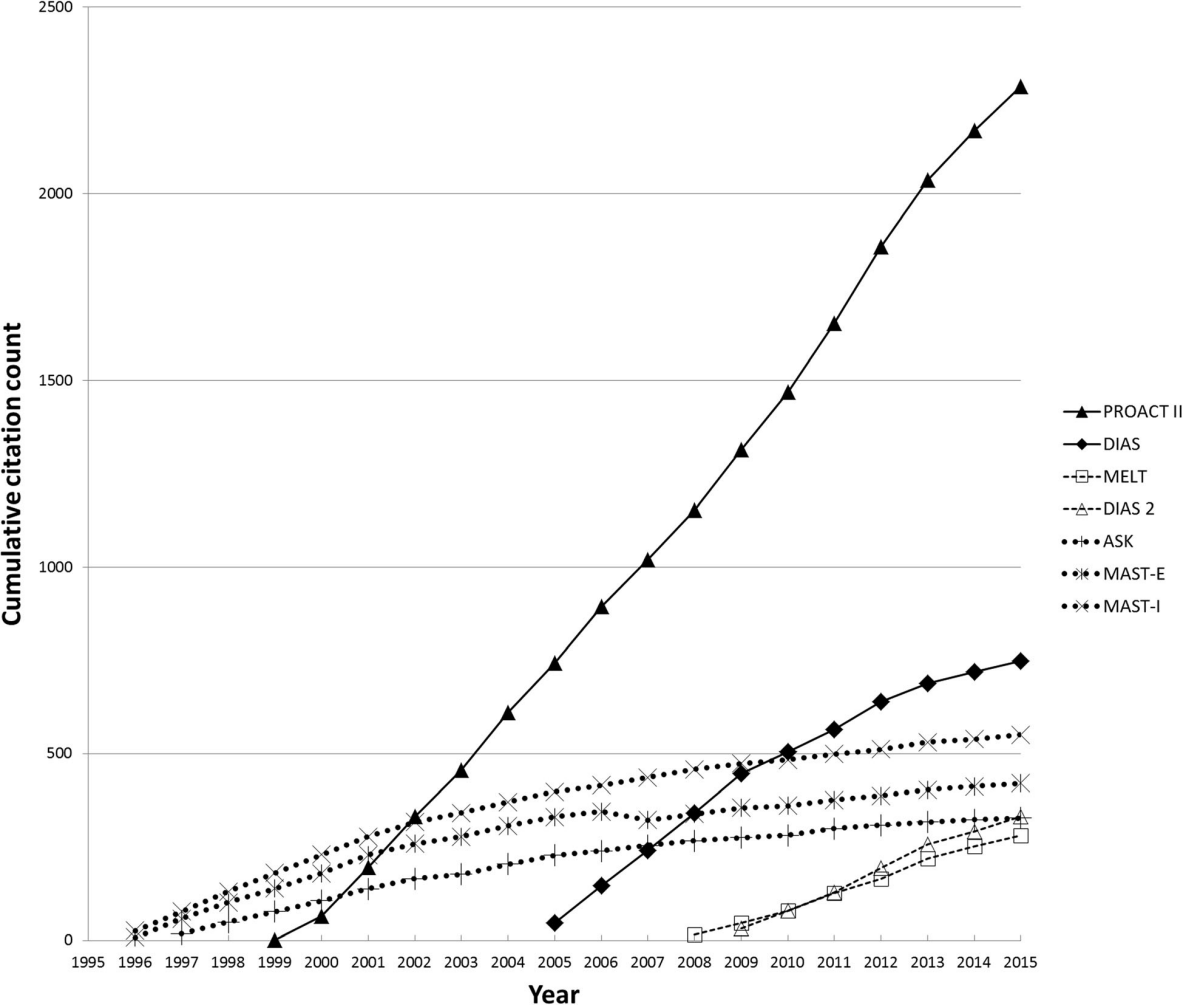
Citation rates of non-alteplase (non-tPA) thrombolytic trials for acute stroke. Cumulative citation counts over time for positive (*solid lines*), *neutral dashed lines*), and negative (dotted lines) randomized controlled trials of thrombolytic agents other than tPA for acute ischemic stroke

Seven trials that met the inclusion criteria involved thrombolytic agents other than tPA, including streptokinase, urokinase, prourokinase, and desmotoplase. Two were positive (PROACT II [19], DIAS [20]), two were neutral (DIAS-2 [21], MELT [22]), and three were stopped early for harm (ASK [23], MAST-E [24], MAST-I [25]). The positive trials received a mean of 112 citations per year, the neutral trials received a mean of 41 citations per year, and the negative trials received a mean of 22 citations per year (Fig. 2).

**Fig. 3:**
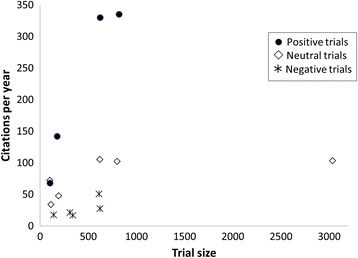
Relationship between sample size and citation rates of thrombolytic trials. Number of study participants and mean annual citation count for trials of thrombolytic agents for ischemic stroke, classified according to trial results

All of the included tPA trials had at least 100 participants. A total of 1445 participants were enrolled in the positive trials of tPA, 4556 participants in neutral trials, and 755 participants in negative trials. There was no statistical relationship between the number of participants in the trial and the number of citations per year (*r* = 0.056; *p* = 0.90). For any given trial size, trials with positive results had equal or more citations per year than trials with neutral results, and trials with neutral results had more citations than trials with negative results (Fig. 3).

## Discussion

We analyzed citation rates from eight major trials of intravenously administered tPA and seven major trials of other thrombolytic agents for the treatment of acute ischemic stroke. Among the tPA trials, those showing a statistically significant benefit from tPA were cited more than three times as often as neutral trials and nearly 10 times as often as negative trials. Positive trials involving other thrombolytic agents were also cited far more frequently than neutral or negative trials. These results demonstrate the presence of substantial citation bias.

While the factors influencing individual author decisions about what studies to cite are likely quite varied, the net effect of these various influences with respect to tPA and other thrombolytics is to disproportionately cite positive trials relative to neutral and negative trials. Over time, this phenomenon has the potential to distort the medical literature in several important ways. First, if clinicians and policy-makers do not utilize a systematic approach when reviewing the medical literature on a given topic they are likely to be exposed to a biased subset of all the potentially relevant trials. Second, as the number of MEDLINE-indexed publications continues to grow, the sheer number of included manuscripts will make it more and more difficult to perform comprehensive medical literature searches based on keywords alone. As a result, readers are increasingly likely to utilize citation webs as part of their medical literature search strategies, potentially exacerbating any existing citation bias. For example, the ranking algorithm from Google Scholar puts great weight on the number of citations [26]. Additionally, citation counts are often used as markers of the quality and impact of published research, further emphasizing the results of highly cited, positive trials as compared to those with fewer citations [27, 28].

When possible, preferential citation of high-quality systematic reviews which utilize and report meticulous search methods may help to limit citation bias due to the selective citation of individual trials. In particular, meta-analyses using individual patient data allow for assessments of both efficacy and safety outcomes with applicability beyond the confines of sometimes homogeneous individual trial populations [29, 30].

Additionally, it is likely that journal impact factor has a causal influence in the citation rates of the included trials, and we considered adjusting our results based on impact factor. However, there is also a likely causal relationship between the direction of study results and the publishing journal [31]. Therefore, rather than functioning as a confounder, in this case impact factor is more likely to be a mediator between the direction of trial results and citation rate. As a result, adjusting for impact factor would be expected to result in an underestimation of the relationship between trial results and citation rate. For this reason, we did not adjust our analysis for impact factor of the publishing journals, though our Table 1 lists the identity of the publishing journal for each included trial.

Several limitations of this work should be considered when interpreting these results. First, classification of trials according to comparisons based on the primary outcome measure is not always straightforward. For example, the DIAS trial had multiple intervention groups and multiple primary endpoints; we classified it as positive because several of the analyses assessing these primary endpoints showed results favoring one or more of the intervention groups [20]. Similarly, the ATLANTIS B trial was stopped early, with no between-group difference in the primary efficacy outcome. However, rates of intracranial hemorrhage, symptomatic intracranial hemorrhage, and fatal intracranial hemorrhage were significantly higher in the intervention group; there was a strong trend towards increased overall 90-day mortality in the intervention group (*p* = 0.09); and in their conclusion the study authors describe their results as “negative” [18]. Based on these factors we classified the trial as negative.

An additional limitation is that we were unable to assess the context in which citations were made. For example, inclusion of a citation does not always mean that the cited study is presented favorably, and to some extent a high citation rate might be a marker of controversy rather than influence. Similarly, we did not assess in which journals studies were cited nor did we evaluate references to studies in the lay press; some journals have more influence on clinicians, researchers, and policy than others and summaries of studies in the lay press may also influence the impact of a study. Finally, while many of the included trials were similar with respect to sample size, publication date, and publication in a high-impact journal, it is likely that other study characteristics, including geographic location and author group, also influenced citation rates. Importantly, however, it is unlikely that the timing of tPA administration alone explains the observed differences in citation rate. For example, the IST-3 trial enrolled more patients in the 0–3-hour window than the NINDS trial [11, 16], and more patients in the 3–4.5-hour window than the ECASS III trial [12], yet the citation curve for IST-3 is consistent with the curves of the other neutral trials rather than the curves observed for these two positive trials. Likewise, the enrollment windows for the ECASS III trial (3 to 4.5 hours) [12] and the ATLANTIS B trial (3 to 5 hours) [18] were similar, yet the annual citation rate for ECASS III is over six times that of ATLANTIS B.

## Conclusions

Reducing citation bias requires vigilance on the part of authors, editors, and peer reviewers but the available evidence suggests that citation bias is rarely identified during the review process [32]. Routine reliance on systematic literature reviews, when possible, may help to limit citation bias, though more work is needed to develop and test approaches which reduce the selective citation of medical research.

## Additional file

Additional file 1: STROBE Statement—checklist of items that should be included in reports of observational studies. (DOCX 35 kb)

## Abbreviations

ASK: Australian Streptokinase Trial
DIAS: Desmotoplase in Acute ischemic Stroke
ECASS: European Cooperative Acute Stroke Study
EPITHET: Echoplanar Imaging Thrombolytic Evaluation Trial
IST: International Stroke Trial
MAST-E: Multicentre Acute Stroke Trial – Europe
MAST-I: Multicentre Acute Stroke Trial – Italy
MELT: Middle Cerebral Artery Embolism Local Fibrinolytic Intervention Trial
NINDS: National Institute of Neurological Disorders and Stroke
PROACT: Prolyse in Acute Cerebral Thromboembolism Trial
tPA: Alteplase
